# On-Device Learning of Indoor Location for WiFi Fingerprint Approach

**DOI:** 10.3390/s18072202

**Published:** 2018-07-09

**Authors:** Marco Aurelio Nuño-Maganda, Hiram Herrera-Rivas, Cesar Torres-Huitzil, Heidy Marisol Marín-Castro, Yuriria Coronado-Pérez

**Affiliations:** 1 Information Technology Department, Universidad Politécnica de Victoria, Ciudad Victoria 87130, Mexico; hherrerar@upv.edu.mx (H.H.-R.); 1439005@upv.edu.mx (Y.C.-P.); 2 Information Technology Laboratory, Cinvestav-Tamaulipas, Ciudad Victoria 87130, Mexico; 3 Cátedras CONACYT, Autonomous University of Tamaulipas, Ciudad Victoria 87000, Mexico; hmarin@conacyt.mx

**Keywords:** mobile application, classifier, WiFi fingerprint, indoor localization

## Abstract

Indoor positioning is a recent technology that has gained interest in industry and academia thanks to the promising results of locating objects, people or robots accurately in indoor environments. One of the utilized technologies is based on algorithms that process the Received Signal Strength Indicator (RSSI) in order to infer location information without previous knowledge of the distribution of the Access Points (APs) in the area of interest. This paper presents the design and implementation of an indoor positioning mobile application, which allows users to capture and build their own RSSI maps by off-line training of a set of selected classifiers and using the models generated to obtain the current indoor location of the target device. In an early experimental and design stage, 59 classifiers were evaluated, using data from proposed indoor scenarios. Then, from the tested classifiers in the early stage, only the top-five classifiers were integrated with the proposed mobile indoor positioning, based on the accuracy obtained for the test scenarios. The proposed indoor application achieves high classification rates, above 89%, for at least 10 different locations in indoor environments, where each location has a minimum separation of 0.5 m.

## 1. Introduction

Nowadays, there is a great demand for applications that are able to provide localization-based services, and in the Internet of Things era, it will be increasingly important to accurately and efficiently locate an object in the real world. The location information plays an important role in various applications such e-health, environment monitoring and home office automation, among others. Localization is a process of ascertaining the location of unknown nodes in a network with the help of some reference nodes called anchors or beacon nodes [1]. On the other hand, wireless technologies have entered the realms of consumer applications, as well as medical, industrial, public safety, logistics and transport systems along with many other applications. Self-organizing sensor networks, location-sensitive billing, ubiquitous computing, context-dependent information services, tracking and guiding are some of the numerous possible application areas. Since wireless information access is now widely available, there is a high demand for accurate positioning in wireless networks [2]. An Indoor Positioning System (IPS) is a framework consisting of a network of devices (both customized or off-the-shelf) that are used to wirelessly locate objects or people carrying handheld devices inside a building where outdoor positioning techniques face serious problems to operate properly [3]. Even when outdoor positioning has shown success in different applications domains, thanks to the use of global navigation satellite systems, indoor positioning is still an open and challenging problem to be solved. In [4], a study of how different environments, structures, equipment, people and even climates affect each and every technique differently is reported.

In [5], a detailed taxonomy of wireless indoor positioning systems is given. Basically, there are two main categories: device-based localization and device-free localization. The former is addressed in this work as we are interested in smartphone-based indoor localization, due to its increasing application-driven demand and commodity access. In device-based localization, there are two subcategories: one is smartphone-based, where a wireless device is attached to the target and computes its localization through cooperation with other deployed wireless devices. Some of the device-based localization techniques are based on 2D models, which combine the signal intensity with positioning algorithms. In this category, devices utilize Bluetooth, ZigBee and WiFi as positioning signals. The second subcategory is tag-based positioning, which is based on 3D models, where infrared, Ultra-Wideband (UWB) and ultrasonic signals are used to compute positioning. Common positioning methods in this subcategory include TOA (Time Of Arrival method), AOA (Angle Of Arrival method) and other positioning methods based on time and space attributes. One disadvantage of tag-based positioning is the additional hardware required when compared with smartphone-based positioning. Related to a high-level application of indoor localization, in [6], a multidimensional taxonomy of Internet-based Indoor Navigation (IIN) services is proposed.

On the other hand, there are some commercially available systems on the market such as the IndoorAtlas [7], which only uses the interference of the magnetic field of the Earth generated by construction and metal objects to estimate the location of a mobile device. The authors in [8] present another method based on magnetic field feature extraction from magnetic field signatures, which is improved through a feature selection process using a Genetic Algorithm (GA) approach. The results reported in this work point toward a reduction of the features required for indoor location estimation. In spite of magnetic field localization being promising, the accuracy of the reported results is not clearly better than other technologies utilized for IPS. Furthermore, they require technologies that have not yet been integrated in the latest WiFi standards. Thus, fingerprint-based positioning still remains an active area of research because of its potential to be applicable using off-the-shelf technologies.

There are many fingerprint-based positioning systems and architectures that try to resolve the problem of determining location. They vary widely in many parameters including accuracy, cost, size, reconfigurability, security and reliability. Wireless Local Area Networks (WLAN), using the 802.11b standard (WiFi), provide local wireless access to network architectures, and thanks to the availability of these networks throughout buildings increasing, they provide a potentially exploitable infrastructure for indoor positioning. WLANs can be used for positioning by converting measurements of the Received Signal Strength (RSS) into a classification problem, using machine learning classifiers. The biggest problem for exploiting the availability of WLANs is the signals’ instability, which concerns localization accuracy, since the propagation of radio signals is complex: range, variations, diffraction, reflection, distortion and scattering are the main obstacles to achieving accuracy. Normally, positioning can be conducted only in places where several Access Points (AP) cover the whole area of interest, so that a position can be inferred at any place and time [9,10].

Indoor positioning based on WiFi signals is inexpensive and widely available, and it does not require external markers; however, the techniques utilized are computationally greedy, and it is difficult to reach real-time performance on mobile devices. One traditional approach for enabling machine learning on smartphone devices consists of having a dedicated server where data captured on the device are sent, then the training is performed on a server, where the models are created from stored data. Later, a device sends to the dedicated server new data to be classified by querying the models located on the server. This approach is very convenient where there is a good network infrastructure, but sometimes, it is not possible to use it in most places where wireless network infrastructure is limited. This approach has been applied to several tasks, such as speech recognition, image recognition, object recognition and gesture recognition, among others, due to the resource-limited nature of mobile devices.

Following this machine learning approach for positioning, Bagosi [10] proposed a system to locate a robot indoors. The algorithms K Nearest Neighbors (KNN), weighted KNN, weighted centroid and the Gaussian kernel method were implemented and compared. The weighted centroid method provided the lowest accuracy; the 5-KNN and the 5-WKNN methods showed an average error rate of 1.5 m; while the Gaussian method outperformed all the others with error rate values under 1 m. The MapUme [11] positioning system is based on a client-server architecture. The client collects the signal strength data and delivers them to the server. The server combines all the information of the locations in a unique and discrete fashion via Bayesian filtering and uses a sequential Monte Carlo method to estimate the location. This system provides an average of 1.6 m of error in the experiments performed. Yu-Chung Cheng et al. in [12] compared a suite of Wireless-Radio-Based (WRB) positioning algorithms to understand how they can be adapted for ubiquitous deployment with minimal calibration. The authors proposed a positioning infrastructure to maximize coverage across entire metropolitan areas. It collects data by wardriving, a process in which WiFi beacons map the location of access points with the help of WiFi and GPS-equipped computers. Data are continuously collected by a variety of users as they move throughout the region.

The main advantage of this system is that it can work also outdoors, achieving an accuracy in the range of 13–40 m. Teemu Roos et al. in [13] compared the nearest neighbor, kernel and histogram methods. The test area consisted of a one-floor office with normal environmental conditions. The training data were collected twice within five days, by using a 2-m grid, with 20 observations. With a short history, the probabilistic methods were more accurate than the nearest neighbor method, while with the full history, the accuracy was approximately the same. In [14], a WiFi fingerprinting method for low-speed vehicle localization using a raw data smoothing technique using an ensemble classification neural network was proposed. In [15], the combination of hierarchical localization and fuzzy classifier ensembles applied to indoor localization was reported. In [16], bagging and the combination of bagging with random subspace were applied to train J48G-based MCSs (fast decision trees) and FURIA-based MCSs (fuzzy rule-based classifiers) devoted to dealing with the WiFi localization problem, which was addressed as a high dimensional classification problem. In [17], the authors presented an enhanced machine-learning localization scheme incorporating AP selection and signal strength reconstruction based on RSSI measurements. In [18], a confidence measure, which is capable of reflecting the uncertainty of the positioning predictions and adjusting the size of the prediction set accordingly, was proposed. In [19], the problem of IPS was explained from the perspective of GPS signals. However, GPS signals are not able to penetrate most construction materials, so the greater part of the world’s commerce, being conducted indoors, cannot be followed by GPS satellites. This is one of the first works related to IPS using radio-frequency identification techniques, which has served as a basis for several works published later. A summary of the reviewed technologies is shown in Table 1.

A comparison in terms of hardware cost, algorithm complexity, user convenience and power-consumption is shown in Table 2. The hardware cost criterion is based on the elements required for the acquisition of RSSI in some cases. The algorithm complexity criterion is based on the techniques utilized for indoor localization. Some of them are neural networks, support vector machines, probabilistic methods and fuzzy methods, among others. Most of the techniques show high accuracy results, but they are computationally expensive. Related to the user convenience criterion, most of the techniques have not been implemented on smartphones or do not allow a final user to experiment directly with the proposed model. Finally, for the power consumption criterion, it is difficult to compare systems implemented on different technologies (PC, compact PC, smartphone, etc.), but the comparison was based on the platform utilized for the final application.

It is important to point out that collecting WiFi data is a difficult task, and some research is related to how to automatize this process. In [28], a fast path-based fingerprint collection mechanism for site survey was proposed. In [29], an indoor localization system that captures user-generated walking trajectories augmented with sensor readings, clusters them and merges them into a map was proposed. On the other hand, alternative techniques to supervised learning have been explored. In [30], the authors proposed a semi-supervised learning algorithm that requires minimal user intervention, but the main disadvantage or this approach consists of the requirement of a server where samples are sent in order to compute the indoor location. Another approach is Simultaneous Localization and Mapping (SLAM), where a system must have the ability to function in situations where map information or current positions are initially unknown or partially unknown and where environment modifications are possible. SLAM has proven to be an effective method for indoor navigation [31,32].

In this paper, an Indoor Positioning Mobile Application (IPMApp) to estimate the indoor location of a smartphone using pre-trained classifiers is proposed. Different classifiers were trained and evaluated within the Weka toolkit [33] in order to compare their accuracies and select a subset of classifiers with high accuracy. The selection of fingerprint technology was motivated by solving the localization problem inexpensively by collecting signal strength samples over the whole localization area. Selected classifiers are evaluated and integrated into the IPMApp and then further tested on different devices and scenarios. An extensive experimental evaluation is provided to validate the performance of the proposed smartphone-based indoor localization, in terms of high classification rates and adaptation to the 10 different locations.

The main contribution of this paper is the design and implementation of a mobile application that serves as an RSSI indoor capture device, that allows the final user to capture and store a set of RSSI lecturesfor any number of locations and at the same time that can be used for training and generating a model that can be useful for estimating the indoor location based on the RSSI lectures available in the area of interest. The rest of the paper is organized as follows. In Section 2, the methodology used to develop the proposed IPMApp is presented. In Section 3, experiments, results and discussions are reported. Finally, conclusions and future work are presented in Section 4.

## 2. IPMApp Design

In this section, the design of the IPMApp is described. Before going into details, the scenarios where RSSI data are captured, which include places such as a residential home and an office buildings, are briefly described.

### 2.1. Proposed Scenarios

**Figure 1:**
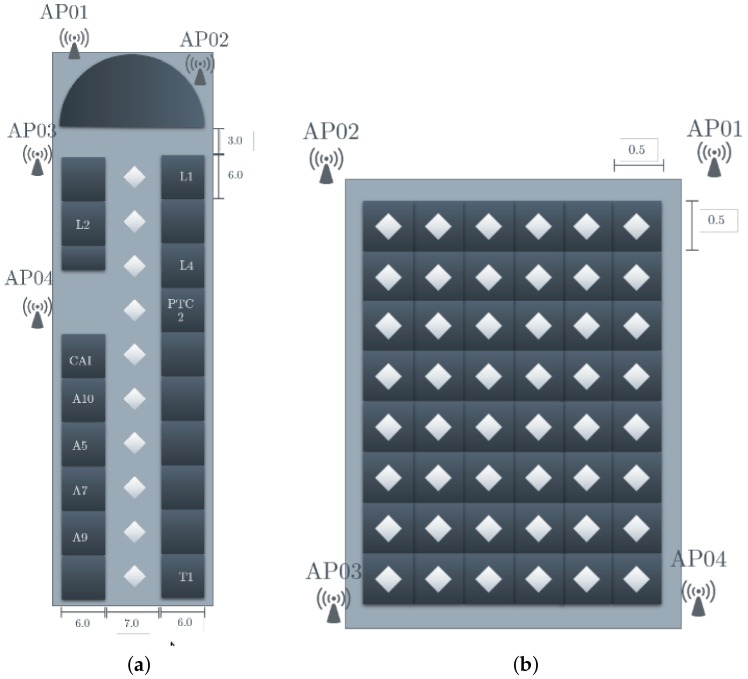

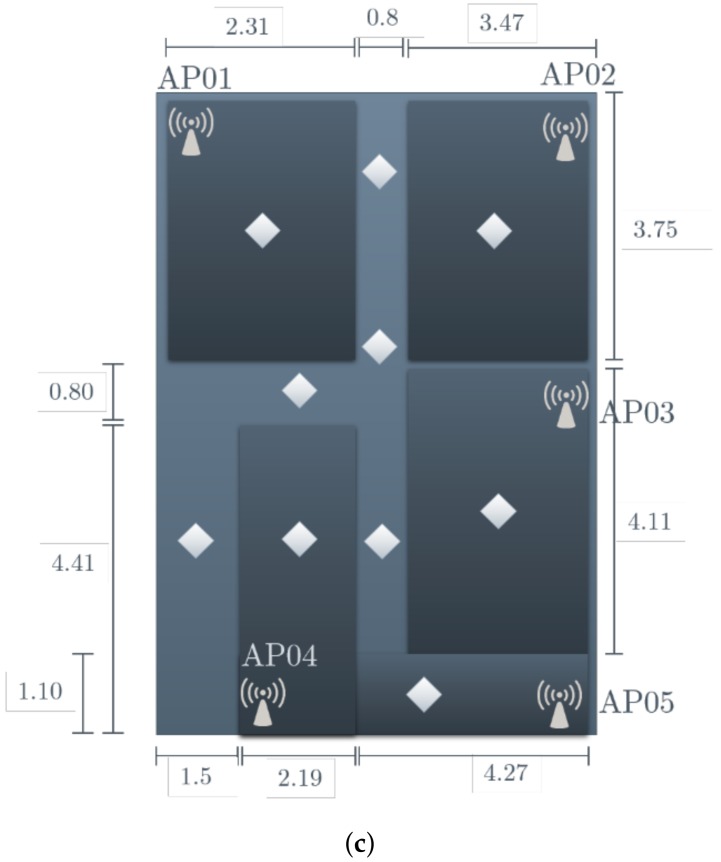
Testing scenarios for the proposed Indoor Positioning Mobile Application (IPMApp). (**a**) Second floor of the main building of the UPV: first scenario; (**b**) Postgraduate hall floor plan: second scenario; (**c**) Residential home floor plan: third scenario.

A previous step for the design of the IPMApp consisted of the selection or design of the test scenarios for validation. The first scenario was the second floor of the main building of the UPV university, which is composed of classrooms and office rooms. The floor plan of this scenario is shown in Figure 1a. In this scenario, 10 locations were captured, each one with a 6-m separation among them. The second scenario was a large office, named the Postgraduate Hall. The floor plan of this scenario is shown in Figure 1b. In this scenario, 48 locations were captured, each one with a 0.5-m separation among them. Finally, the third scenario was a residential home, located outside of the campus. In his scenario, 10 locations were captured, but with different separations due to the home design. The floor plan of this scenario is shown in Figure 1c. The AP legends in Figure 1 stand for Access Point, and the diamonds represent the scenario’s location where the smartphone was located for capturing the RSSI of this specific area.

### 2.2. Modules of the Proposed IPMApp

**Figure 2:**
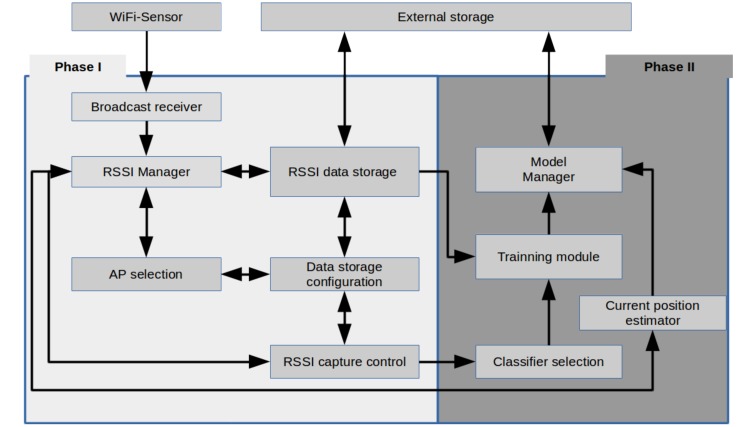
Block diagram of the proposed application for IPMApp.

In Figure 2, the block diagram of the conceptual design of the proposed IPMApp is shown. The modules of the proposed IPMApp are grouped into two major parts. The first one is the Phase 1 block, integrated with modules that perform functions for reading RSSI from WiFi, storing the obtained signals in ARFFfiles or a previously-recorded dataset from existing ARFF files. The modules contained in the Phase 1 block are used to automatically capture RSSI data, which are used for evaluating classifiers in order to decide which ones are going to be included in the final version of the IPMApp. The modules of this phase are briefly described below:

- Broadcast Receiver (BR): This module interfaces directly with the WiFi adapter, in order to determine which APs are available and to obtain information of the RSSI of each AP.
- RSSI manager: This module obtains the list of available APs from the BR, and for each one of the detected APs, the signal strength is obtained.
- AP selection: This module allows the user to select which APs will be used to capture a set of lectures. This module sets the value of AP variable depending on the total APs selected by the user.
- Data Storage Configuration (DSC). This module allows the user to configure the number of locations (LO), the number of lectures (LE) and the name of the ARRF file to store the data.
- RSSI data storage: This module allows one to read and write ARRF files to external storage. When the user wants to create a new dataset, this module takes as input the ARRF name provided by the DSC module and creates a file in the device’s external storage. When the capture process finishes, an ARRF file with LO and LE per location, with AP + 1 columns, is generated, where the number of locations is selected by the user in each lecture column representing the signal strength of the each selected AP. When the user wants to load an existing dataset, this module takes as input the ARFF name, reads its contents and generates a data structure available to other modules. This module also performs the format conversion to normalize the received RSSI.
- RSSI capture control. This module allows the user to control when to start the capturing process in each location and generates an auditive signal when the lectures of a specific location have been captured, so the user can move to the following location to continue with the capture process.

The second part of the IPMApp is the Phase 2 block, integrated with modules that perform functions for generating the models from ARRF files and using the generated models or previously generated models to estimate the indoor position. The modules of this phase are described below:

- Classifiers’ selection: This module allows the user to select which classifiers are going to be used for generating the models.
- Training module: This module allows the user to start the training process, which generates the models to be used for the indoor localization estimation.
- Module manager: This module creates an appropriate data structure in memory for hosting information about the generated models and manages the saving and loading models from external storage.
- Current position estimator: This module computes the device’s indoor position based on the RSSIs received from the RSSI manager, using the models generated and trained previously by the user.

## 3. Experimental Setup and Results

### 3.1. Hardware Tools

- Mobile device with wireless adapter: In this device, the developed application was deployed, and several tests were performed in order to capture datasets for the proposed scenarios, as well as to validate the indoor localization estimation. The proposed application has been validated on smartphone devices with several Android versions. In Table 3, a list of devices and the specifications used for testing and evaluation of the proposed application are shown. The application worked without problems in the listed devices.
- Personal computer for training the models: A desktop computer with an Intel M Quad Processor (T7250@2.66 GHz), 4 GB DDR2 RAM and the Ubuntu 14.01 LTS operating system (64-bit version) was used only in the early stage for the evaluation of all 59 classifiers.

### 3.2. Software Tools

The mobile application was developed using Android Studio IDE with the Android Studio SDK and Java SE Development Kit. The modules of the application were written in Java. In addition, Weka tools were integrated with the application as an external library in order to generate and use models for indoor localization on the device.

### 3.3. Screens of the Developed IPMApp

**Figure 3:**
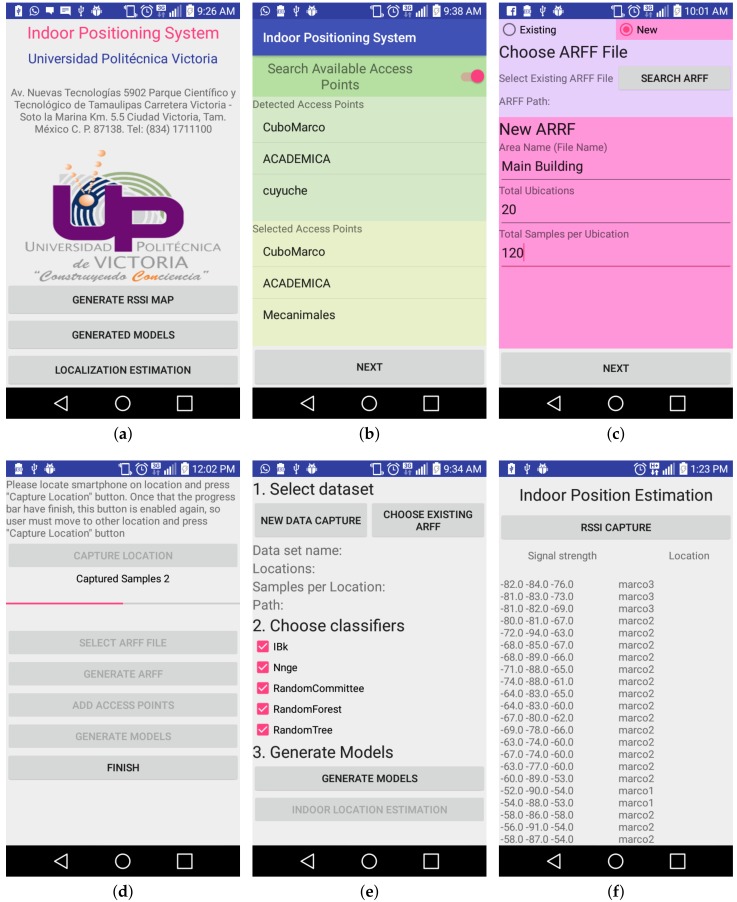
Screen of the proposed IPMApp application. (**a**) Main screen; (**b**) Access point selection; (**c**) Configuration of captured dataset; (**d**) RSSI signal capture; (**e**) Training of ML classifiers; (**f**) Real-time indoor position estimation.

In Figure 3a, the main screen of the IPMApp is shown. There are three main functionalities available for the user. The first one consists of capturing data from the selected device; the second one allows the user to train a model using data previously captured; and the third one consists of using models previously trained to get the location. Each functionality is launched by a button located on the IPMApp main screen.

In the screenshot shown in Figure 3b, the user is asked to select the access points required for generating the RSSI map. Two list are displayed: the first one contains the available APs. As soon as the user selects an AP, this is stored in the second list. One restriction of this module is that at least three APs must be selected in order to continue to the capture and training steps.

In Figure 3c, a screenshot for setting basic configurations of data acquisition is shown. It is required to input a name for an ARFF file when a new capture is started, or it is possible to use one existing ARFF file, and the samples collected in this phase are appended to the existing selected file. In this screen, also the number of localizations and the number of samples to be captured per localization must be settled.

In Figure 3d, a screenshot for controlling the data collection is shown. The application shows a start capture button, a progress bar and other UI controls. The user must be located in the first location and press the start capture button. When the capture starts, the button is disabled, and the progress bar moves from left to right depending on the capture progress. Once the capture for one specific location ends, the start button is enabled, and the user must move to the next location and start the capture process again. When all the locations to be captured have been, the rest of the buttons are enabled for moving to the future steps. This screen can be launched from the RSSI signal capture screen (Figure 3c) or from the main screen (Figure 3a).

In Figure 3e, a screenshot for starting the training is shown. In the first step, it is possible to select the ARFF to be used as the training file. In the second step, it is possible to select the models to be trained, and when the user presses the Generate Models button, the training starts.

In Figure 3f, a screenshot for estimating the real-time indoor localization is shown. When the Estimate Location button is pressed, the process for capturing data from previously-selected APs and estimating the indoor location is started. This screen can be launched from the training screen (Figure 3d) or from the main screen (Figure 3a).

### 3.4. Off-Line Classifier Evaluation

Using data captured by the modules contained in Phase 1 of the IPMApp, an off-line process is executed in order to determine which classifiers achieve higher accuracies. In this step, data obtained from the proposed scenarios were used to train 59 different classifiers, by programming a script in BASH (Bourne Again Shell), feeding the data to the classifiers and running the classifiers sequentially in the batch file. For this purpose, a two-fold cross-validation was utilized, and the mean accuracies of 100 executions of the top-12 classifiers for the three dataset of the proposed scenarios are shown in Table 4.

**Figure 4:**
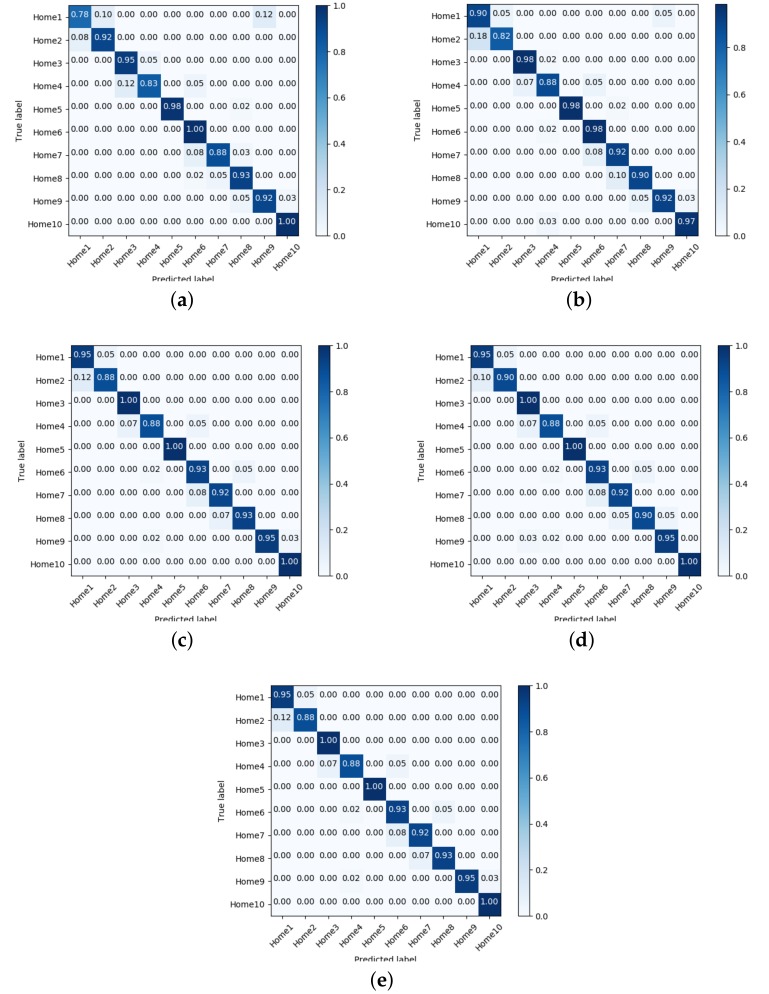
Confusion Matrices for the top-five classifier in Phase 1. (**a**) NNge; (**b**) Ibk; (**c**) Random Committee; (**d**) Random Forest; (**e**) Random Tree.

In order to reduce the number of classifiers, the confusion matrices for the top-five classifiers in Phase 1 were obtained. These matrices are shown in Figure 4. From the confusion matrices, it can be observed that there are minimal variations on locations far from the main diagonal, which helps to infer that possible misclassified samples are of contiguous regions instead of far regions. The top-five selected classifiers are: NNge, Ibk, random tree, random forest and random committee.

Once the final classifiers are ranked and selected, it is possible to perform data capture of the place where indoor localization is desired. In the case that an ARRF file contains an exceptional number of RSSI lectures, the on-line generation of the models should be avoided, so that the models can be generated in offline mode in a conventional PC and then transferred to the device’s external storage, to be used in the proposed IPMApp.

The accuracy performance varies due to the testing scenarios utilized for the proposed IPMApp. The scenarios are open and non-controlled, and there was normal pedestrian activity. Most of them are not flat; there are walls and other devices that introduce noise that the proposed IPMApp is able to manage.

One critical factor to be considered for the IPMApp is the number of APs to be considered for training. To guarantee an accurate indoor localization, at least three APs must be considered. In Table 5, the results for indoor location using different numbers of APs are reported. For this experiment, five localizations taken from the third proposed scenario (Figure 1c) were used. For each location, a data capture process was performed using 3, 4 or 5 APs. From the results obtained, it can be concluded that increasing the number of APs is beneficial for most cases; however, in real scenarios, it is not possible to add as many APs as needed. To validate the robustness of the proposed IPMApp, tests using models generated by other devices were performed. This allows concluding that it is possible to create robust models by obtaining similar accuracies when compared to those obtained in the device where data were captured. The results in Table 5 confirm similar accuracy results when the models are used to estimate the indoor positioning in a different device where data were captured.

## 4. Conclusions

In this paper, the design and implementation of a mobile application for indoor positioning is reported. The proposed application allows the user to create his/her own maps using the available wireless Access Points (APs) in a building. Once those data have been captured, it is possible to train a selected group of classifiers for generating models. In order to determinate which classifiers would be integrated with the IPMApp, in an early stage, data from three different scenarios were captured, and 59 classifiers were evaluated, using a two-fold cross-validation. From this early stage, five classifiers were selected due to the high accuracy obtained. These classifiers were included in the IPMApp and were tested in the same proposed scenarios, obtaining at least 89% accuracy during the test phase. From the results obtained, it can be concluded that the IPMApp allows the final user to capture RSSI lectures for training classifiers and performing the indoor localization accurately.

## Figures and Tables

**Table 1:** Relevant works related to several techniques applied for Indoor Positioning Systems (IPSs). WRB, Wireless-Radio-Based.

IPS	Technologies	Algorithms	Accuracy
[12]	WLAN and GPS	WRB	13–40 m
[7,20]	Earth’s magnetic field and WiFi	N.A.	4 m
[21]	WiFi and IMU	MLA	1.96 m
[6,9,10,11,13,14,15,16,17,18,22,23,24,25,26,27,28,29,30]	WLAN and RSSI	Bayesian Filter, ANNs, Gaussian kernels, KNN, among others	1.60 m

**Table 2:** Comparison of existing WiFi-based IPS.

Work	Hardware Cost	Algorithm Complexity	User Convenience	Power Consumption	Requires Calibration
[6]	Low	High	High	High	n/a
[9]	Low	High	High	High	Yes
[11]	Low	High	High	High	No
[13]	High	High	Low	n/a	No
[14]	High	High	Low	High	No
[15]	High	High	High	High	No
[16]	Low	High	Low	High	No
[17]	Low	High	Low	High	No
[18]	Low	High	Low	High	No
[23]	Low	High	Low	High	No
[25]	Low	High	High	High	Yes
[27]	High	High	Low	High	No
[28]	High	High	Low	High	No
[29]	High	High	Low	High	No
[30]	High	High	Low	High	No

**Table 3:** Devices utilized for testing and evaluating the proposed application.

Device	Processor	RAM	Android Version
Galaxy S2	Dual-core 1.2 GHz Cortex-A9	1 GB	4.1
Galaxy S4	Dual-core 1.7 GHz Krait 300	1.5 GB	5.0.1
Polaroid Tab	Dual-core 1.0 GHz Broadcom 21663	1 GB	4.2.2
Galaxy Tab 4	Quad-core 1.2 GHz Marvell PXA1088	3 GB	5.0.2
Galaxy Tab 10.1	Quad-core 2.3 GHz Krait 400	3 GB	5.1.1
LG G3 Stylus	Quad-core 1.3 GHz Cortex-A7	1 GB	5.0.2
Motorola Moto G	Quad-core 1.4 GHz Cortex-A53	1 GB	5.1.1

**Table 4:** Accuracy obtained by the top 12 classifiers.

Classifier	Accuracy
AttributeSelectedClassifier	95.89
Baggin	91.65
BFTree	93.47
ClassBalancedND	94.15
DataNearBalancedND	94.09
Decorat	97.56
DTNB	89.4
END	96.61
FilteredClassifie	89.41
**Ibk**	**97.71**
J48	96.31
J48graft	96.32
Jrip	91.4
Kstar	97.29
NBTre	96.67
ND	93.55
**Nnge**	**98.18**
OrdinalClassClassifier	91.2
PAR	96.62
**RandomCommitte**	**97.64**
**RandomForest**	**97.64**
RandomSubSpace	95.39
**RandomTree**	**97.62**
Ridor	90.83
RotationForest	96.8
SimpleCar	95.65

**Table 5:** Accuracies obtained using different numbers of APs in the third scenario.

APs	C1	C2	C3	C4	C5
3	89.2%	90.9%	85.4%	85.0%	90.0%
4	95.0%	**96.6%**	94.3%	96.0%	94.6%
5	**99.3%**	89.2%	**99.1%**	**99.3%**	**98.0%**
